# Reelin Is Required for Maintenance of Granule Cell Lamination in the Healthy and Epileptic Hippocampus

**DOI:** 10.3389/fnmol.2021.730811

**Published:** 2021-08-13

**Authors:** Catarina Orcinha, Antje Kilias, Enya Paschen, Marie Follo, Carola A. Haas

**Affiliations:** ^1^Experimental Epilepsy Research, Department of Neurosurgery, Medical Center – University of Freiburg, Faculty of Medicine, University of Freiburg, Freiburg im Breisgau, Germany; ^2^Biomicrotechnology, Department of Microsystems Engineering (IMTEK), University of Freiburg, Freiburg im Breisgau, Germany; ^3^Faculty of Biology, University of Freiburg, Freiburg im Breisgau, Germany; ^4^Lighthouse Core Facility, Department of Internal Medicine I, Medical Center – University of Freiburg, Faculty of Medicine, University of Freiburg, Freiburg im Breisgau, Germany; ^5^Center for Basics in NeuroModulation, Faculty of Medicine, University of Freiburg, Freiburg im Breisgau, Germany

**Keywords:** dentate granule cells, hippocampus, granule cell dispersion, epilepsy, Reelin

## Abstract

One characteristic feature of mesial temporal lobe epilepsy is granule cell dispersion (GCD), a pathological widening of the granule cell layer in the dentate gyrus. The loss of the extracellular matrix protein Reelin, an important positional cue for neurons, correlates with GCD formation in MTLE patients and in rodent epilepsy models. Here, we used organotypic hippocampal slice cultures (OHSC) from transgenic mice expressing enhanced green fluorescent protein (eGFP) in differentiated granule cells (GCs) to monitor GCD formation dynamically by live cell video microscopy and to investigate the role of Reelin in this process. We present evidence that following treatment with the glutamate receptor agonist kainate (KA), eGFP-positive GCs migrated mainly toward the hilar region. In the hilus, Reelin-producing neurons were rapidly lost following KA treatment as shown in a detailed time series. Addition of recombinant Reelin fragments to the medium effectively prevented the KA-triggered movement of eGFP-positive GCs. Placement of Reelin-coated beads into the hilus of KA-treated cultures stopped the migration of GCs in a distance-dependent manner. In addition, quantitative Western blot analysis revealed that KA treatment affects the Reelin signal transduction pathway by increasing intracellular adaptor protein Disabled-1 synthesis and reducing the phosphorylation of cofilin, a downstream target of the Reelin pathway. Both events were normalized by addition of recombinant Reelin fragments. Finally, following neutralization of Reelin in healthy OHSC by incubation with the function-blocking CR-50 Reelin antibody, GCs started to migrate without any direction preference. Together, our findings demonstrate that normotopic position of Reelin is essential for the maintenance of GC lamination in the dentate gyrus and that GCD is the result of a local Reelin deficiency.

## Introduction

Reelin is a key regulator of neuronal positioning during mammalian brain development, acting as a possible stopping signal (Dulabon et al., 2000; Chai et al., 2009; Sekine et al., 2011). Additionally, Reelin it is also important for synaptic function, plasticity and memory formation in the adult brain (for review see Herz and Chen, 2006; D’Arcangelo, 2014; Jossin, 2020). During development of telencephalic structures, such as the cerebral cortex and the hippocampus, Reelin is synthesized by early born Cajal-Retzius (CR) cells, later mainly by interneurons (Alcántara et al., 1998; Pesold et al., 1998). Reelin is a large glycoprotein, which is secreted into the extracellular matrix, where it is proteolytically cleaved into smaller isoforms, an important prerequisite for activation of target cells (Jossin et al., 2004, 2007; Tinnes et al., 2011; 2013). Cleavage of Reelin can occur at two sites: N-terminally between the second and the third repeat, and C-terminally between the sixth and seventh repeat, generating five isoforms depending on the protease in action (Lambert de Rouvroit et al., 1999; Jossin et al., 2004; Sato et al., 2016). However, which Reelin fragment is required for the normal Reelin signaling is still a matter for debate. Some research shows that the so-called central Reelin fragment, consisting of repeats 3–6 (R3-6), is imperative for receptor activation (D’Arcangelo et al., 1999; Jossin et al., 2004; Lee and D’Arcangelo, 2016), whilst other results demonstrate that the N-terminal (Nakajima et al., 1997; Dulabon et al., 2000) or C-terminal (Nakano et al., 2007) portion of the Reelin molecule is significant.

The canonical Reelin pathway involves binding of Reelin to lipoprotein receptors, the very-low-density lipoprotein receptor (VLDLR) and the apolipoprotein E receptor 2 (ApoER2), leading to tyrosine phosphorylation of the intracellular adaptor protein Disabled-1 (Dab1). Since VLDLR and ApoER2 lack core kinase activity, this is compensated for by the recruitment of Src family kinases. It results in the subsequent activation of downstream effectors, which target the actin and microtubule cytoskeleton (D’Arcangelo et al., 1999; Howell et al., 1999; Tissir and Goffinet, 2003; Stolt and Bock, 2006; Jossin and Goffinet, 2007; Leemhuis and Bock, 2011; Bock and May, 2016). Recently, it was reported that Reelin also interacts genetically and biochemically with ephrin/EphB receptors (Sentürk et al., 2011; Bouché et al., 2013). Ephrin/EphB and Reelin signaling share several downstream effector molecules and there is considerable overlap in the processes regulated by ephrinB/EphB and Reelin, showing that these molecules help mediate Reelin signaling outcomes (reviewed in Niethamer and Bush, 2019).

Dysregulation of Reelin signaling has been associated with several brain disorders such as autism, schizophrenia, bipolar disorder, depression, Alzheimer’s disease and epilepsy (reviewed in Ishii et al., 2016; Devinsky et al., 2018; Armstrong et al., 2019). The pathoetiologies of these conditions have been associated with aberrant brain cytoarchitecture, impaired synapse formation and stability and improper neuronal migration, most likely caused by a loss of Reelin. False neuronal positioning is particularly observed in mesial temporal lobe epilepsy (MTLE), typified by recurrent focal seizures and Ammon’s horn sclerosis (Thom, 2014). The latter is characterized by neuronal loss and often by granule cell dispersion (GCD), a malpositioning of dentate granule cells (GCs) (Houser, 1990; Haas et al., 2002).

There is evidence that a loss of Reelin is involved GCD formation, since the Reelin-deficient *reeler* mouse (D’Arcangelo et al., 1995; Hirotsune et al., 1995) shows a disorganized granule cell layer (GCL), reminiscent of GCD (Frotscher et al., 2003). Moreover, GCD formation has been shown to be accompanied by a loss of Reelin-producing neurons in the hippocampus of MTLE patients (Haas et al., 2002; Liu et al., 2020) and in rodent epilepsy models (Heinrich et al., 2006; Gong et al., 2007; Antonucci et al., 2008; Duveau et al., 2011; Orcinha et al., 2016). A local rescue of GC lamination has been achieved *in vivo* by infusion of recombinant Reelin into the mouse hippocampus during epileptogenesis (Müller et al., 2009) and *in vitro* by addition of recombinant Reelin in kainate (KA)-treated organotypic hippocampal slice cultures (OHSC; Orcinha et al., 2016). Conversely, neutralization of Reelin in the healthy mouse hippocampus by infusion of the function-blocking CR-50 antibody caused a local widening of the GCL (Heinrich et al., 2006). GCD can be induced *in vitro* in OHSCs by KA application (Tinnes et al., 2011, 2013; Chai et al., 2014). In this *in vitro* model, differentiated GCs have been shown to migrate via somal translocation (Murphy and Danzer, 2011; Chai et al., 2014), but so far the precise mechanism has remained unclear.

In the present study, we used KA-treated OHSC from transgenic mice expressing enhanced green fluorescent protein (eGFP) in differentiated GCs (1) to monitor the movement behavior of individual GCs in detail by live cell video microscopy, (2) to investigate which part of the Reelin molecule is efficient in GCD rescue, (3) whether the site of the Reelin loss matters, and (4) whether Reelin is needed for the maintenance of lamination in the healthy hippocampus. We present evidence that differentiated GCs actively migrate toward the Reelin-free hilar region and this motility can be prevented by application of the recombinant N-terminal or the central Reelin fragment. In addition, we show that placement of Reelin-coated beads into the hilus significantly stops the aberrant migration of GCs after KA exposure, and that neutralization of Reelin by the CR-50 antibody causes locomotion of GCs in healthy OHSC.

## Materials and Methods

### Animals

Experiments were performed with transgenic Thy1-eGFP mice, in which eGFP is expressed under the control of the Thy1 promoter in a subpopulation of differentiated GCs in the dentate gyrus (RRID: IMSR_JAX:007788, M-line, C57BL/6 background, Feng et al., 2000). Mice were bred at the Center for Experimental Models and Transgenic Service (CEMT), University of Freiburg, and kept in a 12 h light/dark cycle at room temperature (RT) with food and water *ad libitum*. All animal procedures were handled according to the guidelines of the European Community’s Council Directive of 22 September 2010 (2010/63/EU) and were approved by the regional council (Regierungspräsidium Freiburg).

### Organotypic Hippocampal Slice Cultures (OHSC)

Seven- or eight-days-old (P7-P8) male and female Thy1-eGFP mouse pups were used for OHSC preparation as described previously (Gerlach et al., 2016; Orcinha et al., 2016). In brief, brains were rapidly removed from the skull after decapitation under isoflurane (Abbott) anesthesia. The hippocampi were dissected and sliced (400 μm) perpendicular to the longitudinal axis of the hippocampus using a McIlwain tissue chopper. Only slices from the mid portion of each hippocampus were used. The slices were placed onto culture inserts (Millicell cell culture inserts, RRID:SCR_015799, Merck) and transferred to 6-well plates with 1 mL of incubation medium (pH 7.2) per well containing 50% minimal essential medium (MEM, Gibco), 25% basal medium Eagle (BME, Gibco), 25% heat-inactivated horse serum (Gibco) supplemented with 0.65% glucose (Braun) and 2 mM glutamate (Gibco). OHSC were incubated as static cultures (Stoppini et al., 1991) in 5% CO_2_ at 37°C for at least 7 days *in vitro* (DIV) to allow full eGFP expression (Chai et al., 2014) which was checked using an inverted epifluorescence microscope (Olympus CKX41 with U-RFL-T, Olympus). Incubation medium was changed every second day.

### Live Cell Imaging

Organotypic hippocampal slice cultures were prepared as described above and cultivated for 7 – 18 DIV for the different live cell imaging experiments. Immediately before imaging, OHSC were handled as follows: (1) to monitor the migration behavior of individual GCs, OHSC were treated with KA (10 μM; Tocris) for 45 min, followed by addition of fresh medium with or without recombinant Reelin fragments (1 nM); Reelin fragments were produced as previously described in Orcinha et al. (2016); (2) to study the influence of Reelin in normotopic position within the dentate gyrus, OHSC were treated with KA (10 μM) for 45 min followed by addition of fresh medium, and placement of Reelin-coated fluorescent microspheres into the hilus (see below); and (3) to neutralize endogenous Reelin, the monoclonal CR-50 antibody (8 μg/mL; MBL Int. Corp) or normal mouse IgG (8 μg/mL; Santa Cruz) was added to fresh medium. In all control experiments, OHSC were cultured in fresh medium, only.

For live cell imaging, a laser scanning confocal microscope (Zeiss LSM 880) was used equipped with a multiline Argon 488 nm scanning laser. OHSC were securely placed in a stage enclosed in an aerated chamber at 37°C with humidified atmosphere containing 5% CO_2_, and were imaged along the *z*-axis with a spacing of 7 μm using a 20× objective (Plan Apochromat NA 0.8), at 45 min intervals over a period of 8 h (stack acquisition between each interval took roughly 15 min). The lowest laser power setting was used to avoid photo bleaching and associated photo damage. At the end of the imaging period, the confocal z-stacks obtained for each time point were converted to a maximum projection image using the Zen 2 lite software (Zeiss) and exported as TIFF-files for further analysis.

### Analysis of Migration Behavior

To assess the migration pattern and direction of individual eGFP-positive GCs, a custom Matlab^®^ software script (Matlab R2017b, RRID:SCR_001622, The-MathWorks) was applied. The confocal image stacks obtained at the different time points were imported into the analysis script and individual eGFP-positive GCs were automatically detected and numbered (Supplementary Figures 1A,B). Only cells with a diameter of approximately 10 μm and visible throughout all raw images were flagged by the function trackCells in the custom script. Taking into account the scaling factor (conversion from pixel to μm), cell motility was quantified by calculating (1) the total path length (Supplementary Figure 1C, totalDist) over 8 h and (2) the effective migration distance (Supplementary Figure 1C, effectDist) as the length of the resulting vector between start and end point (Supplementary Figure 1C, start and finish). These values were also used to calculate the directionality ratio as the quotient of effective distance and total path length according to Gorelik and Gautreau (2014) (Supplementary Figure 1C). If the neuron had a straight trajectory, this ratio was close to 1.0 (0.80 – 1.0). If the values were smaller (<0.80), a frequent change in direction had occurred. To analyze the migration direction, the border between hilus and GCL (white dotted line in Supplementary Figure 1A) or between microspheres and GCL (white dotted line in Supplementary Figure 1B) were marked and the distance of the start and end point to the hilus/microsphere – GCL border was calculated. Cells with a reduced distance to the border [H_*distance*_(*t* = 0 h) – H_*distance*_(*t* = 8 h)] were considered to migrate toward the hilus, whereas cells with an increased distance in relation to the hilus-GCL/microsphere border moved toward the molecular layer (ML).

### Preparation of Reelin-Coated Fluorescent Microspheres

For the application of Reelin into the hilus of OHSC, red fluorescent latex beads (10 μL; Lumafluor Inc.) were incubated with recombinant full-length (FL) Reelin (1 nM) overnight at 4°C with mild agitation. Then, microspheres were centrifuged, the supernatant was removed, and the remaining beads were resuspended in 5 μL of sterile saline as previously described (Dulabon et al., 2000). A 0.8 nL bead solution (uncoated or Reelin-coated) was placed into the hilus of each OHSC using a programmable nanoliter injector (Nanoject III, Drummond) with a glass pipet fixed to a micromanipulator. Due to the broad bandwidth of the fluorophore used to produce the red beads, it was possible to detect them easily during the live cell imaging experiments using the green channel (see Supplementary Figure 2).

### Immunohistochemistry

For the time course analysis of Reelin expression following KA application, OHSC were treated with KA (10 μM) for 45 min followed by incubation in fresh medium. Slices were fixed at 0 (immediately after KA treatment), 1, 2, 3, 4, 6, and 8 h post-KA with 4% paraformaldehyde (PFA, Roth) in 0.1 M phosphate buffer (PB; pH 7.4; 4 h at RT) and subsequently rinsed several times in PB. Immunolabeling for Reelin was performed using a free-floating protocol (Orcinha et al., 2016). After pre-treatment (0.25% Triton X-100, 10% normal serum in PB, 2 h at RT), slices were incubated (0.1% Triton X-100, 1% normal serum in PB, 24 h at RT) with a mouse monoclonal anti-Reelin antibody (G10, 1:1.000, Millipore, Cat# MAB5364, RRID: AB_2179313). Antibody binding was visualized by incubation with a goat anti-mouse Cy5-conjugated secondary antibody (1:400, RRID: AB_2338714, Jackson ImmunoResearch Laboratories) in the dark (6 h at RT) and counterstained with DAPI (4′,6-diamidino-2-phenylindole; 1:10.000, Roche). Whole slices were dried on glass slides, coverslipped with anti-fading mounting medium (DAKO, Sigma Aldrich, United States) and stored in the dark at 4°C.

### Quantification of Reelin-Immunofluorescence in OHSC

Immunolabeled slices were analyzed with an epifluorescence microscope (Axio Imager 2 with ZEN blue software, Zeiss) and photomicrographs were taken with a 10-fold objective (Plan Apochromat, NA 0.45). Exposure times were kept constant to allow comparison between individual slices. To quantify fluorescence intensity of Reelin signals in the hilus, photomicrographs were converted to grayscale and the signal intensity was quantified as integrated density using the ImageJ software (ImageJ, RRID: SCR_003070). The regions of interest were manually outlined with the polygon selection tool. Values were corrected by background subtraction: integrated density – (measured area × mean background signal). Background was measured in each picture in a small region close to the area of interest and without Reelin signal. The mean background was calculated for each experiment and for the area of the hilus.

### Western Blot Analysis

Eight – ten OHSC per animal and treatment were pooled and placed in 200 μL of lysis buffer (50 mM Tris pH 8.0, 150 mM NaCl, 1% Triton, supplemented with protease inhibitor cocktail cOmplete^TM^ and phosphatase inhibitor tablets PhosSTOP^TM^ (Roche Diagnostics). The samples were homogenized, the protein suspension was shaken for 15 min at 4°C and the insolubilized fraction was removed by centrifugation at 12,500 rpm for 10 min at 4°C. The supernatant was collected, and protein concentration was quantified using the Bicinchoninic Acid Assay (BCA) kit (Pierce). The protein concentration was determined following the instructions described in the manual. For the colorimetric measurement, the absorbance was read at 562 nm using bovine serum albumin (BSA) as standard.

For electrophoresis, 20 μg of total protein per lane were mixed with loading buffer. The samples were size-fractionated by standard sodium dodecyl sulfate – polyacrylamide gel electrophoresis (NuPAGE^TM^, 4–12% Bis-Tris gel, Invitrogen) and transferred to polyvinylidene difluoride membranes (Roche Diagnostics). Membranes were blocked with I-Block (Tropix) or 5% BSA (for phosphorylated cofilin) in Tris-buffered saline with Tween-20 (TBST) for 1 h, washed with TBST and incubated overnight at 4°C with the following primary antibodies: rabbit polyclonal anti-Dab1 (1:1.000; Abcam), rabbit monoclonal anti-GAPDH (1:10.000; Clone 14C10; Cell Signaling) and rabbit polyclonal anti-p-cofilin (Ser3) (1:1.000; Cell Signaling). After several washing steps, the membranes were incubated with goat anti-rabbit alkaline-phosphatase-conjugated secondary antibody (1:10.000; Tropix) for 1 h at RT or overnight at 4°C and CDP Star (Tropix) was used as substrate for chemiluminescent detection by the Fusion FX Western Blot Imaging System (Vilber Lourmat).

Quantification of Western blot signals was attained with ImageJ software and GAPDH density was used as the loading control. In brief, images were opened with the ImageJ software and the color was inverted so that the bands turned black. The bands of interest were marked with the rectangle tool and the band intensity was measured with the “Gels” function available in the software. The band intensity peaks were plotted and the peaks were separated using the line tool. The area under each peak was measured and the size of the peak was indicated as percent of the total size of all of the highlighted peaks. The peak percentage of each analyzed protein was divided by the peak percentage of the respective loading control to give the relative percent of expression for each protein and each condition. These values were normalized to the control band and the relative value for the control group was always set as 100%. The experiments were repeated at least three times for each condition.

### Statistical Analysis

All values were expressed as mean ± standard deviation. All statistical analysis was performed with GraphPad Prism 7 software (RRID: SCR_002798). Differences between groups were tested for statistical significance (Unpaired student’s *t*-test or one-way ANOVA with Tukey’s multiple comparison test). Significance levels were set to ^∗^*P* < 0.05, ^∗∗^*P* < 0.01, ^∗∗∗^*P* < 0.001.

## Results

### Migration Behavior of eGFP-Positive Granule Cells in OHSC After KA Treatment

**FIGURE 1:**
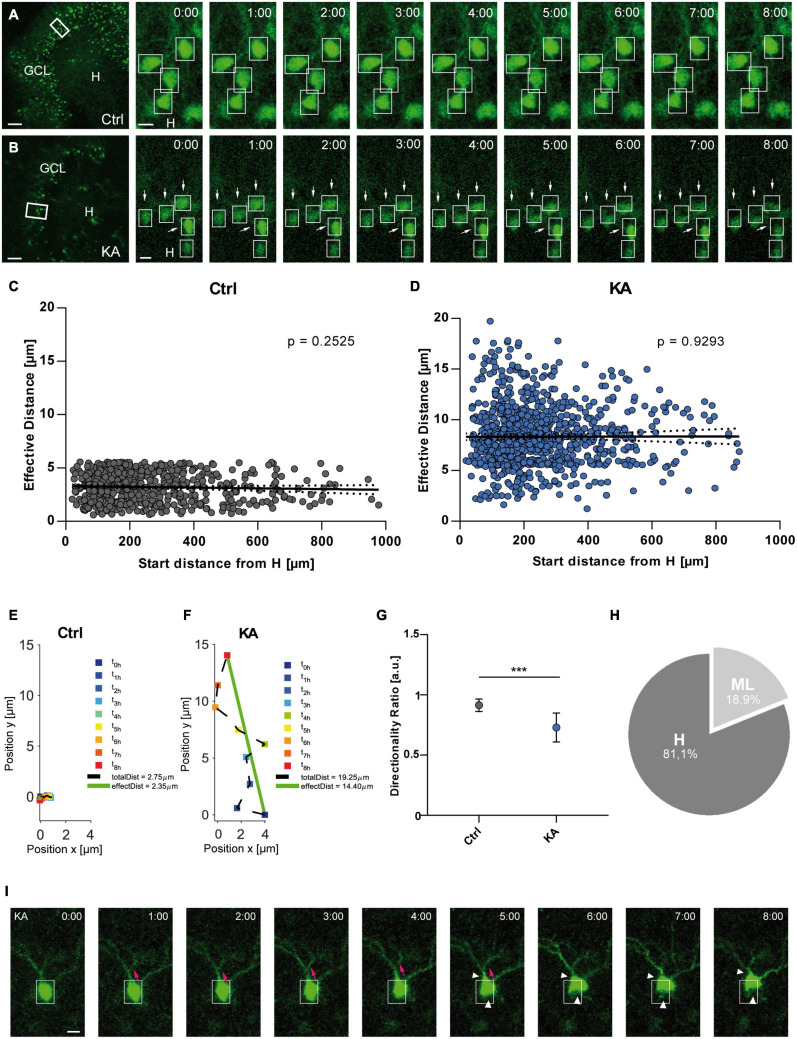
EGFP-positive GCs migrate preferentially toward the hilus after KA treatment. Live cell imaging of individual eGFP-labeled GCs in control OHSC and after exposure to KA over a period of 8 h. **(A,B)** Representative confocal micrographs of nine imaging time points are shown for each condition. Left panel: overview; white frame indicates the area shown at high magnification on the right. Tracked cells are marked by white frames. **(A)** Untreated control. GCs do not change position. **(B)** Treatment with 10 μM KA for 45 min. GCs with high motility are indicated by arrows. Scale bars: 80 μm for overview; 10 μm for high magnifications. **(C,D)** Quantitative evaluation of migration distances of individual GCs in controls (**C**; *n* = 23 slices, 548 cells) and in KA-treated OHSC (**D**; *n* = 20 slices, 868 cells). Each slice was from a different animal. In contrast to controls **(C)**, almost all GCs change their positions after KA treatment **(D)** and travel mainly toward the hilus regardless of their initial position in the GCL. **(E,F)** Graphs showing the representative behavior of an individual, tracked neuron in a control **(E)** and a KA-treated OHSC **(F)**. Cell motility was assessed by calculating both the total length of the traveled path over 8 h (black dashed line = total distance) and the effective distance of the migration as the length of the resulting vector (green solid line) between start (*t* = 0 h) and end point (*t* = 8 h). Note the meandering way of migration in the KA group. **(G)** Diagram showing the directionality ratio (defined as ratio of total to effective distance). GCs from KA-treated slices have a lower directionality ratio than controls since they undergo requent changes in direction. **(H)** Relative distribution of GCs traveling toward the hilus (81.1%) or molecular layer (18.9%) in KA-treated OHSC. Unpaired student’s *t*-test (***P < 0.001). **(I)** Morphological changes following KA application. Representative GC from a KA-exposed culture imaged for 8 h. There is a clear change in the shape of the soma (white arrow heads) and migration of the soma into a dendritic branch (magenta arrows). Scale bar: 5 μm. GCL, granule cell layer; H, hilus; Ctrl, control.

First, we investigated the migration behavior of individual eGFP-positive GCs in KA-challenged OHSC in real time by life microscopy. To this end, OHSC were prepared from Thy1-eGFP mice, in which eGFP is expressed exclusively in differentiated dentate GCs (Chai et al., 2014; Orcinha et al., 2016) and were cultured for 7 days. OHSC were exposed to 10 μM KA for 45 min, followed by incubation in fresh medium and live cell imaging with a laser scanning confocal microscope (Figures 1A,B). Confocal stacks were acquired every hour over a period of 8 h and were evaluated offline by a custom script. The individual eGFP-positive GCs were automatically detected throughout the GCL and their position was tracked with respect to a manually drawn border between the hilus and GCL over the whole observation period (Supplementary Figure 1). The differences in motility were quantified by calculating (1) the total length of the traveled path over 8 h, (2) the effective distance of migration as the length of the vector between start (*t* = 0 h) and end point (*t* = 8 h), and (3) the directionality ratio (see in detail in “Materials and Methods”).

Granule cells showed a significantly higher motility after KA treatment (*n* = 20 slices, 868 cells) when compared to control conditions (*n* = 23 slices, 548 cells) (Figures 1A,B), evident from an increased effective distance (in μm: controls: 3.065 ± 1.358; KA: 8.075 ± 2.975; unpaired *t*-test: *P* < 0.001). In contrast to controls (Figure 1C; Pearson correlation coefficient *r* = −0.04558; ns with *P* = 0.2525), almost all analyzed GCs migrated after KA treatment regardless of their position within the GCL (Figure 1D; Pearson correlation coefficient *r* = 0.00288; ns with *P* = 0.9293). We also found different populations of GCs: some traveled longer distances than others and a small population of neurons did not change their position. Analysis of the migration behavior revealed that GCs in KA-treated OHSCs did not move straight ahead, but were meandering, indicated by a reduced directionality ratio (0.7295 ± 0.1201) (Figures 1E–G). If a neuron has a straight trajectory, this ratio is close to 1.0 (0.8 – 1.0), if there are frequent changes in direction, the ratio is below 0.8. In addition, many GCs (81.1%) in KA-treated slices migrated in the direction of the hilus, whereas only a minority (18.9%) headed toward the molecular layer (Figure 1H). The constant, small values of effective distance in control OHSC were interpreted as adjustments to the environment.

Interestingly, the soma shape of migrating GCs was altered as the movement progressed over the 8 h after KA treatment (Figure 1I). The soma of the GCs appeared to move up toward one dendritic branch (magenta arrows, Figure 1I), then shifted to another one by retraction of the cell body (white arrow heads, Figure 1I), gradually displacing the soma. GCs in control slices did not show any morphological changes.

### Time Course of Reelin Expression in OHSC Following KA Treatment

**FIGURE 2:**
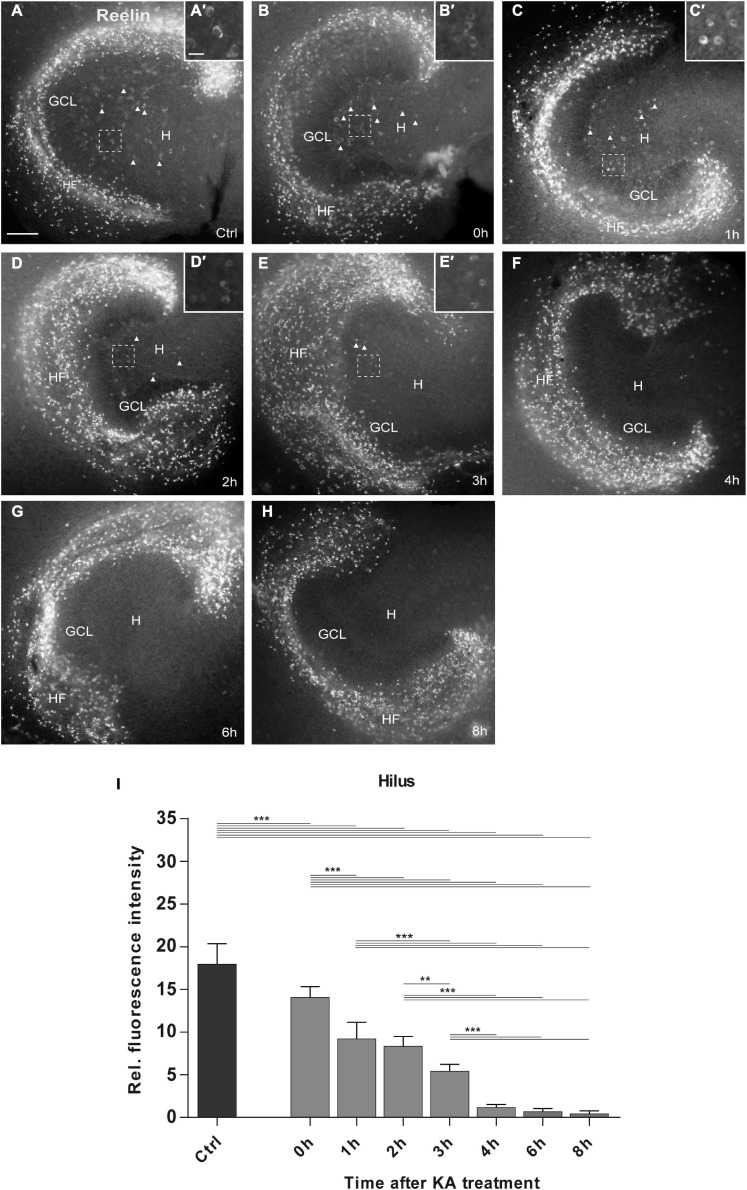
Time course of KA-induced loss of Reelin-expressing neurons in the hilus. Representative images of whole OHSC immunolabeled for Reelin are shown. Small insets show high magnification of Reelin-expressing interneurons in the area of the hilus indicated by the dashed white frame **(A’–E’)**. **(A)** In control slices (*n* = 21 slices), many Reelin-positive Cajal-Retzius cells are present at the hippocampal fissure, and large Reelin-immunopositive interneurons **(A’)** in the hilus (arrow heads). **(B–H)** Slices were fixed at **(B)** 0 h (=45 min after KA) (*n* = 6 slices), **(C)** 1 h (*n* = 7 slices), **(D)** 2 h (*n* = 8 slices), **(E)** 3 h (*n* = 9 slices), **(F)** 4 h (*n* = 9 slices), **(G)** 6 h (*n* = 5 slices), and **(H)** 8 h (*n* = 9 slices) after KA application. Reelin-positive cells are completely lost in the hilus at 4 h after treatment, whereas Reelin-expressing neurons at the hippocampal fissure survive throughout all time points. Note the morphological change of the Reelin-expressing interneurons in the hilus between 0 and 3 h **(B’–E’)** which appear shrunken. **(I)** Densitometric quantification of the Reelin signal in the hilus in controls and after KA treatment. Note the fast and continuous decline of the Reelin signal within the first 3 h after treatment. One-way ANOVA, followed by Tukey’s Multiple Comparison (**P < 0.01; ***P < 0.001). GCL, granule cell layer; H, hilus; HF, hippocampal fissure. Scale bar: 100 μm for overview, 20 μm for insets.

To probe the role of Reelin loss in the migratory behavior of GCs following KA exposure, we performed a detailed time course analysis of Reelin expression in OHSC following KA treatment. As before, OHSC were exposed to 10 μM KA for 45 min and were immediately fixed with PFA (0 h time point) or incubated in fresh medium followed by fixation at 1, 2, 3, 4, 6, and 8 h post-KA treatment. Slices were immunolabeled for Reelin and signals were quantified by densitometry (Figure 2). Control slices were handled in parallel for the corresponding time points, but since the Reelin signal was consistent in all controls, data were pooled (Figure 2A).

In control OHSC, many brightly labeled Reelin-positive neurons were present at the hippocampal fissure (HF) and less abundantly distributed in the hilus, consistent with previous reports (Tinnes et al., 2011; Orcinha et al., 2016). In KA-treated OHSC, many Reelin-immunolabeled neurons remained at the hippocampal fissure, but in the hilus the number of Reelin-positive neurons decreased rapidly between 0 and 8 h post-KA treatment when compared with controls (Figures 2B–H). Densitometric evaluation revealed a significant loss of the Reelin signal in the hilus already at the end of the KA treatment (0 h), which progressed over the next 3 h. After 4 h, all Reelin-immunolabeled neurons had disappeared from the hilus and no significant differences were found between 4, 6, and 8 h after KA exposure: controls: 17.9 ± 2.36 (*n* = 21 slices); 0 h: 14.07 ± 1.24 (*n* = 6 slices); 1 h: 9.24 ± 1.92 (*n* = 7 slices); 2 h: 8.3 ± 1.15 (*n* = 8 slices); 3 h: 5.44 ± 0.79 (*n* = 9 slices); 4 h: 1.16 ± 0.34 (*n* = 9 slices); 6 h: 0.72 ± 0.32 (*n* = 5 slices); 8 h: 0.45 ± 0.32 (*n* = 9 slices); ANOVA: *P* < 0.001; Tukey’s post-test: *P* < 0.001; Figure 2I). In parallel, we observed morphological changes in the Reelin-expressing neurons in the hilus, they appeared shrunken right after KA exposure, until the 3 h time point (Figure 2A’–E’).

This detailed time course showed that Reelin-expressing neurons were rapidly lost (within 3 h) in the hilus following KA treatment, whereas Reelin-synthetizing neurons at the hippocampal fissure survived.

### Influence of Recombinant Reelin Fragments on KA-Induced Motility of eGFP-Labeled GCs

So far, we found that in KA-treated OHSC most GCs migrated toward the hilus, where Reelin-expressing neurons were lost within 3 h after KA exposure. If the Reelin loss indeed plays a role in the migration process, addition of exogenous Reelin should influence it. Therefore, we added recombinant Reelin fragments, specifically N-terminal and the central fragment (R3-6, N-R2, N-R6), to the medium after KA treatment and followed GCs by live cell imaging for 8 h.

**FIGURE 3:**
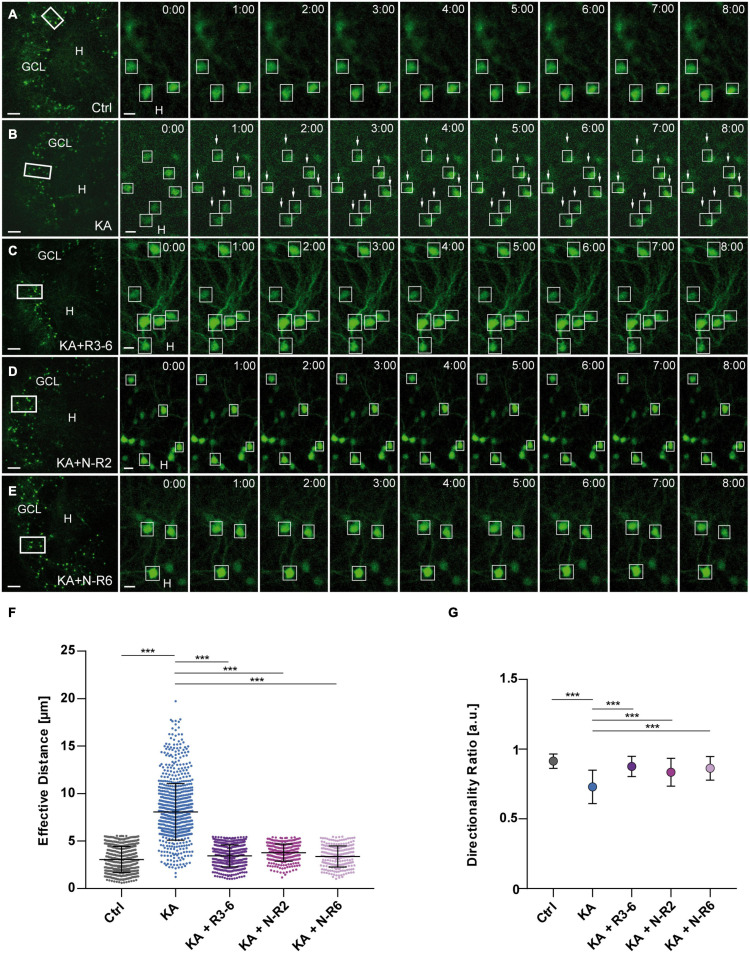
KA-induced migration of differentiated GCs is prevented by recombinant Reelin fragments. **(A–E)** Live cell imaging of individual eGFP-positive GCs was performed over a period of 8 h, and migration distances of automatically selected cells were assessed. Representative confocal micrographs of nine imaging time points are shown for each condition. Left panel: overview; white frame indicates the area shown at high magnification on the right. Tracked cells are marked by white frames. **(A)** Untreated control (*n* = 23 slices, 548 cells). GCs do not change their position. In all experiments, each slice derived from a different animal. **(B)** Treatment of OHSC with 10 μM KA for 45 min (*n* = 20 slices, 868 cells). GCs with high motility are indicated by arrows. **(C–E)** Treatment of OHSC with 10 μM KA for 45 min, followed by incubation with fresh medium and subsequent application of recombinant Reelin fragments (each 1 nM). **(C)** R3-6 (*n* = 12 slices, 470 cells), **(D)** N-R2 (*n* = 9 slices, 284 cells), and **(E)** N-R6 (*n* = 7 slices, 228 cells). There is no migration of GCs in cultures treated with Reelin fragments. **(F)** Quantitative evaluation of migration distances of individual GCs. KA-treated GCs migrate, but migration can be prevented by application of the different recombinant Reelin fragments. **(G)** Diagram showing the directionality ratio. GCs from KA-treated slices show a reduced directionality ratio when compared to controls. Following application of the different recombinant Reelin fragments, directionality ratio of GCs reach control values. One-way ANOVA, followed by Tukey’s Multiple Comparison Test (***P < 0.001; Ctrl vs. KA + R3-6, Ctrl vs. KA + N-R2 and Ctrl vs. KA + N-R6 show no significant difference). GCL, granule cell layer; H, hilus. Scale bars: 80 μm for overview; 10 μm for high magnifications.

As before, KA treatment alone caused a significantly increased motility of eGFP-positive GCs when compared to controls (Figures 3A,B,F). In contrast, GCs, although treated with KA, did not migrate in the presence of recombinant Reelin fragments: R3-6 (*n* = 12 slices, 470 cells), N-R2 (*n* = 9 slices, 284 cells) and N-R6 (*n* = 7 slices, 228 cells) (Figures 3C–F). All three fragments significantly prevented the movement of GCs (in μm: R3-6: 3.448 ± 1.157; N-R2: 3.778 ± 0.9103; N-R6: 3.372 ± 1.108; KA vs. KA+R3-6, KA+N-R2 and KA+NR-6; ANOVA: *P* < 0.001; Tukey’s post-test: *P* < 0.001; Figure 3F). Also, the directionality ratio was increased after incubation with recombinant Reelin fragments (R3-6: 0.869 ± 0.07; N-R2: 0.835 ± 0.10; N-R6: 0.863 ± 0.08) reaching values similar to controls (0.914 ± 0.05), in contrast to slices exposed to KA alone (Ctrl vs. KA+R3-6, KA+N-R2 and KA+NR-6, ns. KA vs. KA+R3-6, KA+N-R2 and KA+NR-6; ANOVA: *P* < 0.001; Tukey’s post-test: *P* < 0.001) (Figure 3G). These results indicate that all applied Reelin fragments were able to stop the KA-mediated migration of eGFP-positive GCs, supporting the role for Reelin as a positional signal for GCs in the hippocampus.

### Placement of Reelin-Coated Beads Into the Hilus of KA-Treated OHSC

Next, we aimed at addressing the question whether the localization of the exogenous Reelin source matters. To this end, we used fluorescent beads with or without recombinant Reelin, placed them with the help of a micromanipulator into the hilus of healthy or KA-treated OHSC and performed life cell imaging for 8 h as described before. Following imaging, individual eGFP-positive GCs were automatically detected in confocal image stacks and their positions were tracked with respect to a manually drawn border between the beads and the GCL (Supplementary Figure 1).

**FIGURE 4:**
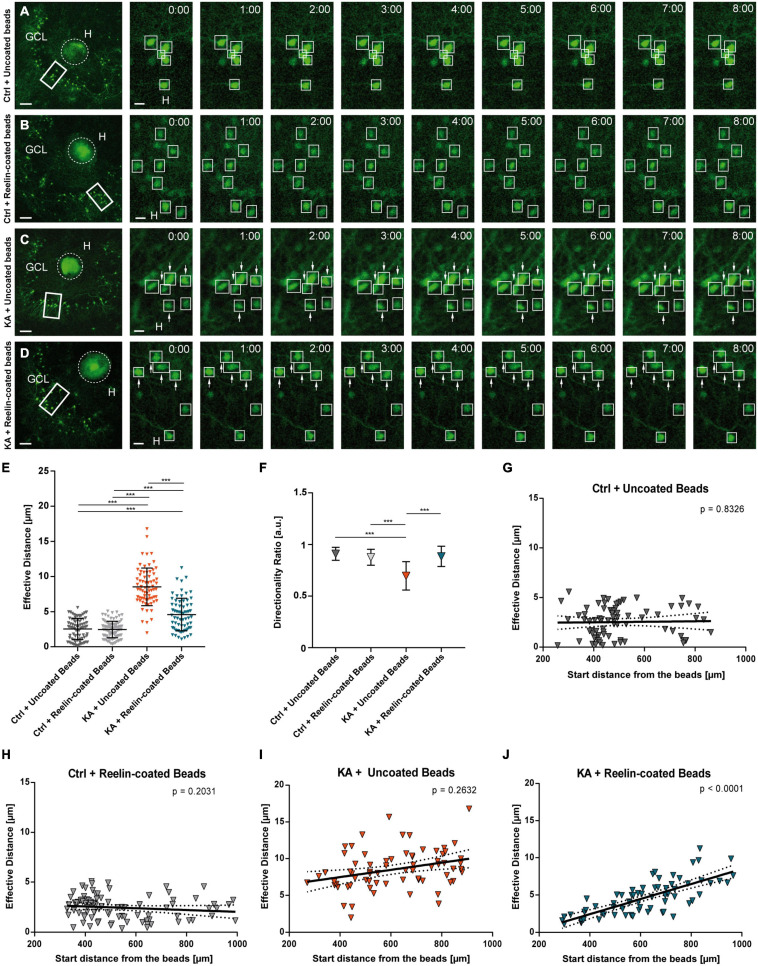
Placement of Reelin-coated beads into the hilus stops KA-induced migration. Live cell imaging of individual eGFP-positive GCs was performed over a period of 8 h, and migration distances of automatically selected neurons were assessed. Cell motility was determined by calculating both the total length of the traveled path over 8 h and the effective migration distance as the length of the resulting vector between start (*t* = 0 h) and end point (*t* = 8 h). The border between the beads and GCL was marked and the distance was calculated between the start and end point of the cells to this border. **(A–D)** Representative confocal micrographs of nine imaging time points are shown for each condition. Left panel: overview; white frame indicates the area shown at high magnification on the right. Tracked cells are marked by white frames and GCs with high motility are indicated by arrows. OHSC were treated with 10 μM KA for 45 min, followed by placement of beads. Uncoated fluorescent beads **(A,C)** or Reelin-coated **(B,D)** were positioned into the hilus. Each slice derived from a different animal **(A)** Control + uncoated beads (*n* = 6 slices, 77 cells). **(B)** Control + Reelin-coated beads (*n* = 7 slices, 89 cells). **(C)** KA + uncoated beads (*n* = 4 slices, 70 cells). **(D)** KA + Reelin-coated beads (*n* = 5 slices, 68 cells). **(E)** Quantitative evaluation of migration distances of individual GCs. Under control conditions with uncoated and with Reelin-coated beads, GCs do not change position. KA induces migration of GCs but this is strongly reduced by placement of Reelin-coated beads. **(F)** Diagram showing the directionality ratios of the different groups. Note that GCs of the KA + uncoated bead group have lower values than the two control groups. GCs of KA-treated OHSC with Reelin-coated beads present a ratio similar to controls. **(G–J)** Quantitative evaluation of migration distances of individual GCs in relation to their start distance from the beads. **(G)** Control + uncoated beads, **(H)** Control + Reelin-coated beads, **(I)** KA + uncoated beads. Note that in these group there is no correlation between effective migration distance and start distance from the beads of tracked GCs. **(J)** KA + Reelin-coated beads. A positive correlation (Pearson correlation coefficient *r* = 0.7461) between the initial position and the migration behavior of the eGFP-positive cells is apparent. One-way ANOVA followed by Tukey’s Multiple Comparison Test (***P < 0.001). GCL, granule cell layer; H, hilus. Scale bars: 80 μm for overview; 10 μm for high magnifications.

First, we assessed whether application of fluorescent beads had any effect when present in control OHSCs (Figures 4A,B). There was no difference in the effective distance of eGFP-positive neurons when uncoated beads (*n* = 6 slices, 77 cells) or Reelin-coated beads (*n* = 7 slices, 89 cells) were placed into the hilus of control slices (Figure 4E) (in μm: uncoated beads: 2.537 ± 1.518; Reelin-coated beads: 2.456 ± 1.178; Ctrl+uncoated beads vs. Ctrl+Reelin-coated beads, ns; Tukey’s post-test: *P* = 0.9937). Moreover, placement of uncoated beads into KA-treated OHSC did not affect the significantly increased motility of GCs (Figures 4C,E) (*n* = 4 slices, 70 cells; 8.527 ± 2.69 μm; Ctrl+uncoated beads and Ctrl+Reelin-coated beads vs. KA+uncoated beads, ANOVA: *P* < 0.001; Tukey’s post-test: *P* < 0.001). Interestingly, when Reelin-coated beads were placed into the hilus of KA-treated OHSC (*n* = 5 slices, 68 cells), the movement of GCs was significantly reduced when compared to GCs from KA-treated slices with uncoated beads (Figures 4D,E) (4.596 ± 2.32 μm; KA+uncoated beads vs. KA+Reelin-coated beads, ANOVA: *P* < 0.001; Tukey’s post-test: *P* < 0.001), but did not reach control levels (Ctrl+uncoated beads and Ctrl+Reelin-coated beads vs. KA+Reelin-coated beads, ANOVA: *P* < 0.001; Tukey’s post-test: *P* < 0.001).

The directionality ratio showed a lower value in GCs of OHSC exposed to KA with uncoated beads (0.695 ± 0.14) when compared with GCs from untreated slices with uncoated beads or with Reelin-coated beads (Ctrl+uncoated beads: 0.909 ± 0.06; Ctrl+Reelin-coated beads: 0.876 ± 0.08; Ctrl+uncoated beads and Ctrl+Reelin-coated beads vs. KA+uncoated beads, ANOVA: *P* < 0.001; Tukey’s post-test: *P* < 0.001). However, in KA-treated OHSC with Reelin-coated beads, the ratio was similar to controls (0.885 ± 0.10; Ctrl+uncoated beads and Ctrl+Reelin-coated vs. KA+Reelin-coated beads, ns; KA+uncoated beads vs. KA+Reelin-coated beads, ANOVA: *P* < 0.001; Tukey’s post-test: *P* < 0.001) (Figure 4F). Moreover, as anticipated, there was no correlation between the effective migration distance and the start distance from the beads in both control groups (Figures 4G,H) and in the KA+uncoated beads group (Figure 4I). In contrast, in KA-treated OHSC with Reelin-coated beads, there was a positive correlation (Pearson correlation coefficient *r* = 0.7461; *P* < 0.001) between the effective migration distance and the initial position from the beads (Figure 4J). This means that GCs closer to the Reelin source did not change their position significantly (values similar to controls) while more distant GCs did, indicating a concentration-dependent effect of Reelin. Apparently, the Reelin-coated beads created a Reelin gradient to which GCs responded in a distance-dependent manner: keeping them in place when sufficient Reelin was sensed but making them move when Reelin concentration was low.

### Effect of KA Application on Intracellular Components of the Reelin Signaling Machinery

Based on the described observations, we aimed at investigating potential effects of KA on the Reelin signal transduction pathway. Dentate GCs express Reelin receptors and the intracellular adaptor protein Dab1 and maintain their expression under epileptic conditions as shown in MTLE patients (Haas et al., 2002) and rodent epilepsy models (Gong et al., 2007; Müller et al., 2009). Activation of the Reelin signal transduction pathway depends on phosphorylation of Dab1 (Howell et al., 1999) and further downstream of cofilin (Chai et al., 2009).

**FIGURE 5:**
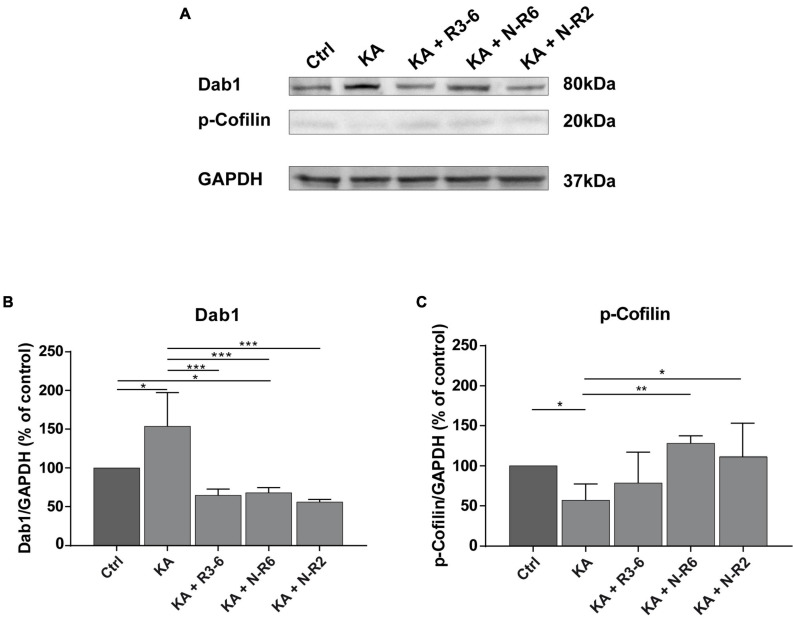
Addition of recombinant Reelin fragments restores KA-mediated alterations of Reelin signaling components. Quantitative Western blot analysis for Dab1, p-cofilin was performed with extracts from control OHSC (*n* = 8 animals, 8–10 slices/animal), OHSC treated with 10 μM KA for 45 min (*n* = 3 animals, 8–10 slices/animal) and OHSC exposed to KA followed by incubation with fresh medium and subsequent application of recombinant Reelin fragments: R3-6 (*n* = 4 animals, 8–10 slices/animal), N-R6 (*n* = 3 animals, 8–10 slices/animal) and N-R2 (*n* = 4 animals, 8–10 slices/animal). **(A)** Representative Western blot for Dab1 and p-cofilin. GAPDH was used as a loading control. **(B,C)** Densitometric evaluation of **(B)** Dab1, **(C)** p-cofilin. Treatment with KA results in a significant upregulation of Dab1 synthesis and reduction of phosphorylated cofilin levels in tissue extracts. Subsequent incubation with the different recombinant Reelin fragments restores Dab1 and p-cofilin levels. One-way ANOVA followed by Tukey’s Multiple Comparison Test (*P < 0.05; **P < 0.01; ***P < 0.001). Molecular weights are shown in kilodalton.

Hence, we investigated intracellular signaling components of the canonical Reelin pathway after KA treatment. OHSC were treated with KA for 45 min, followed by washout, and incubation with or without recombinant Reelin fragments for 8 h. Subsequently, Dab1 and phosphorylated cofilin (p-cofilin) levels were detected by Western blot analysis and densitometrically quantified (Figure 5).

Kainate treatment resulted in significantly elevated Dab1 levels synthesis (% of control: 153.9 ± 43.34; *n* = 3) when compared to controls (Ctrl vs. KA. ANOVA: *P* < 0.001; Tukey’s post-test: *P* = 0.014; *n* = 8). Noteworthy, the incubation with the different Reelin fragments resulted equally in a drop of Dab1 levels, significantly different from KA slices (% of controls: controls: 100%; R3-6 (*n* = 4): 64.76 ± 7.786%; N-R6 (*n* = 3): 67.94 ± 6.63%; N-R2 (*n* = 4): 56.16 ± 3.029%; ANOVA: *P* < 0.001; KA vs. R3-6: Tukey’s post-test: *P* = 0.0005; Ctrl vs. N-R6: Tukey’s post-test: *P* = 0.0007; KA vs. N-R2: Tukey’s post-test: *P* = 0.0002).

The levels of p-cofilin were significantly reduced after KA exposure (% of controls: 57.24 ± 20.17%; Ctrl vs. KA. ANOVA: *P* = 0.0051; Tukey’s post-test: *P* = 0.0452). As shown in Figure 5C, this effect was abolished after the incubation with the recombinant Reelin fragments N-R6 and N-R2, with values similar to controls (controls: 100%; N-R6: 128.2 ± 9.231%; N-R2: 111.2 ± 41.72%; ANOVA: *P* = 0.0051; Ctrl vs. N-R2: Tukey’s post-test: *P* = 0.9434; Ctrl vs. N-R6: Tukey’s post-test: *P* = 0.4598). No difference was found between post-KA treatment and incubation with the central fragment R3-6 (R3–6: 78.69 ± 38.37%; KA vs. R3-6; Tukey’s post-test: *P* = 0.6924).

Together, these findings show that the loss of Reelin post-KA treatment caused an increase of Dab1 and decrease of p-cofilin levels reminiscent of the *reeler* phenotype (Chai et al., 2009) and addition of Reelin to the culture medium reversed these effects.

### Migration Behavior of eGFP-Positive GCs After Neutralization of Reelin

**FIGURE 6:**
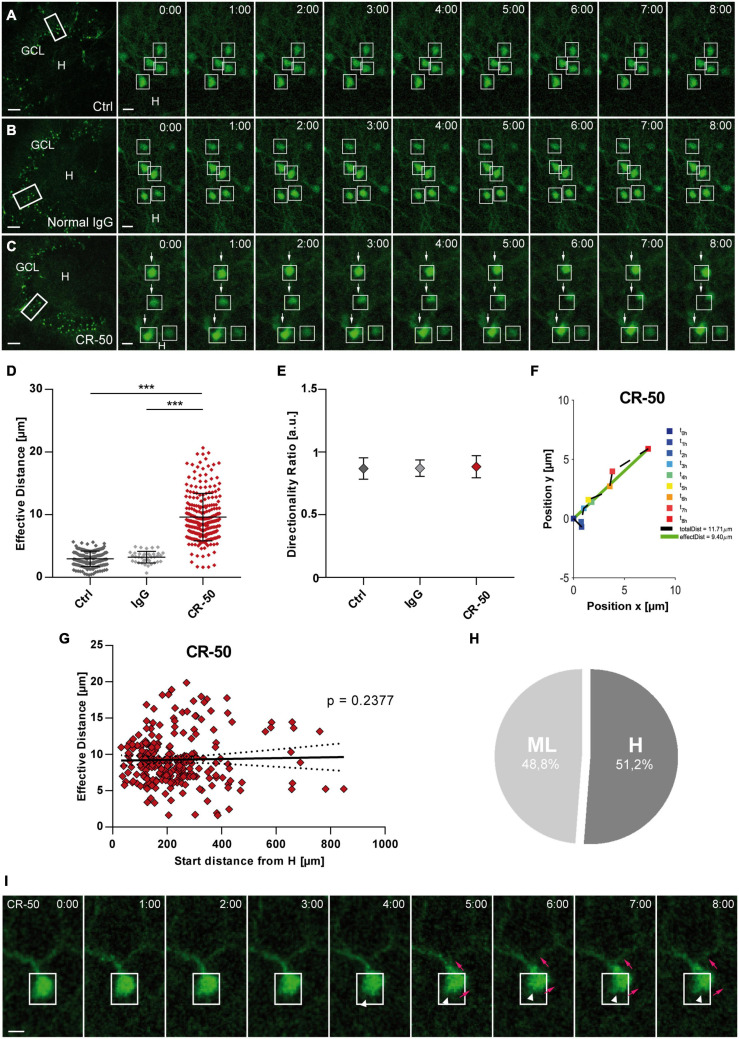
Neutralization of Reelin in the healthy hippocampus leads to migration of mature granule cells. Live cell imaging of individual eGFP-positive GCs was performed over a period of 8 h, and migration distances of automatically selected cells were assessed. **(A–C)** Representative confocal micrographs of nine imaging time points are shown for each condition. Left panel: overview; white frame indicates the area shown at high magnification on the right. Tracked cells are marked by white frames. **(A)** Untreated control. GCs do not change position. **(B)** OHSC incubated with normal mouse IgG (8 μg/mL). As in controls, there is no neuronal displacement. **(C)** OHSC incubated with neutralizing CR-50 antibody (8 μg/mL). GCs with high motility are indicated by arrows. **(D)** Comparison of effective migration distances of individual GCs between groups: controls (*n* = 15 slices; 183 cells), normal mouse IgG (*n* = 2 slices, 38 cells) and CR-50 (*n* = 10; 254 cells). Under control conditions and with normal mouse IgG, GCs do not change position. In contrast, incubation with the CR-50 antibody strongly induces migratory behavior as indicated by strongly increased effective distances. **(E)** Diagram showing the directionality ratio. All three groups show similar values. **(F)** Graph showing the path of one representative neuron after incubation with the CR-50 antibody. Cell motility was assessed by calculating both the total length of the traveled path over 8 h (black dashed line = total distance) and the effective distance of the migration as the length of the resulting vector (green solid line) between start (*t* = 0 h) and end point (*t* = 8 h). Note that the path of this neuron is straight without any changes in direction. **(G)** Graph showing that there is no correlation between effective migration distance and start distance from the hilus of tracked GCs. All cells change their positions and travel regardless of their position in the GCL. **(H)** Relative distribution of cells traveling toward the hilus (51.2%) or molecular layer (48.8%) in OHSC incubated with CR-50 antibody. To differentiate the migration direction, the border between the hilus and GCL was marked and the distance of the start and end point of the cells to this border was calculated Cells that reduce their distance to the border over time [H_*distance*_ (*t* = 0 h) – H_*distance*_ (*t* = 8 h), positive values] are considered migrating toward the hilus. Cells showing an increase in distance (negative values) moved toward the ML. One-way ANOVA, followed by Tukey’s Multiple Comparison Test (***P < 0.001; Ctrl vs. Normal IgG show no significance). Scale bars: 80 μm for overview; 10 μm for high magnifications. **(I)** Representative GC from an OHSC treated with CR-50 and imaged over a period of 8 h, as described above. Note the change in the soma shape as the neuron shifts position (white arrow heads), the soma moves into an apical dendrite (magenta arrows). Scale bar: 5 μm. GCL, granule cell layer; H, hilus.

Finally, we tested the effect of Reelin neutralization in healthy OHSC by application of the function-blocking CR-50 antibody known to block Reelin function *in vivo* and *in vitro* (Ogawa et al., 1995; Nakajima et al., 1997; Heinrich et al., 2006). OHSC from Thy1-eGFP mice were incubated with normal medium (control), normal mouse IgG or CR-50 followed by live cell imaging for 8 h (Figures 6A–C). Normal mouse IgG was used as a negative control to confirm the specificity of the CR-50 antibody and for ruling out non-specific Fc receptor binding (Burry, 2011). As before, individual eGFP-positive GCs were automatically detected throughout the GCL and their position was tracked with respect to a manually drawn border between the hilus and GCL (Supplementary Figure 1).

Granule cells became motile in the presence of CR-50 (Figure 6C) with a significantly increased effective distance when compared to controls (Figure 6D) [in μm: Ctrl: 2.95 ± 1.22 (*n* = 15 slices, 183 cells); IgG: 3.221 ± 0.93 (*n* = 2 slices, 38 cells); CR-50: 9.602 ± 3.78 (*n* = 10 slices, 254 cells); Ctrl vs. IgG, ns with *P* = 0.8564; Ctrl and IgG vs. CR-50, ANOVA: *P* < 0.001; Tukey’s post-test: *P* < 0.001). Interestingly, the directionality ratio was close to 1 (Figure 6E) (Ctrl: 0.869 ± 0.06; IgG: 0.871 ± 0.07; CR-50: 0.883 ± 0.09; ANOVA: ns with *P* = 0.2280) portraying a straight migration behavior in the presence of CR-50 (Figure 6F). No correlation was found between the start distance from the hilus and the effective distance of eGFP-positive neurons after incubation with CR-50. Almost all cells traveled long distances regardless of their initial position in the deep or superficial GCL (Figure 6G) and without any preference in migration direction (Figure 6H). In addition, changes occurred in the soma as the neurons shift position (Figure 6I). As observed in Figure 1I, the soma of the GCs moved up into the apical dendrite (magenta arrows, Figure 6I). In this particular case, there is no retraction of the cell body, only change in shape (white arrow heads, Figure 6I), and the soma continues moving into the dendritic branch. Altogether, these data clearly show that ubiquitous neutralization of Reelin by the CR-50 antibody caused a non-directional migration of eGFP-positive GCs indicating that Reelin is necessary to maintain GC lamination in the healthy hippocampus.

## Discussion

The main findings of the present study are as follows: KA treatment caused a rapid and complete loss of Reelin-producing hilar neurons. Upon KA challenge, eGFP-positive GCs actively migrated toward the Reelin-poor hilar region. This migration was effectively prevented by application of recombinant N-terminal or central Reelin fragments. Placement of Reelin-coated beads into the hilus of KA-treated cultures slowed the migration of GCs in a distance-dependent manner. In addition, we found that KA treatment impaired the Reelin signal transduction pathway by accumulation of Dab1 and reduced phosphorylation of cofilin. Importantly, neutralization of Reelin in healthy OHSC lead to a non-directed movement of GCs underlining the importance of Reelin for maintenance of GC lamination.

### GCs Preferentially Migrate Toward the Reelin-Poor Hilus Following KA Challenge

In the adult dentate gyrus, GCs form a densely packed layer. Under epileptic conditions, the lamination can dissolve resulting in GCD, as observed in MTLE patients (Houser, 1990; Haas et al., 2002; Fahrner et al., 2007) and in the intrahippocampal KA mouse model (Bouilleret et al., 1999; Heinrich et al., 2006; Häussler et al., 2012; Janz et al., 2017). Since neurogenesis is lost in the dentate gyrus following KA injection (Kralic et al., 2005; Heinrich et al., 2006; Nitta et al., 2008; Häussler et al., 2012), it was assumed that differentiated GCs undergo GCD (Chai et al., 2014). Here, we demonstrate in OHSC by live cell microscopy that eGFP-labeled, differentiated GCs become motile in response to KA exposure. The migrating GCs altered their soma shape over the observation period: the nuclei of the GCs moved up toward one dendritic branch followed by a shift to another one, thereby gradually displacing the soma mainly in the direction of the hilus. These observations suggest that the migration of mature GCs described here relies on nuclear movement in line with previous studies describing somal translocation as a novel mechanism of GCs undergoing dispersion (Murphy and Danzer, 2011; Chai et al., 2014). Somal translocation is a mechanism of migratory neurons during brain development, involving the translocation of the nucleus and perisomatic cytoplasm into a leading process (Rakic, 1972; Métin et al., 2008). This migration mode does not require a radial glia scaffold (Miyata et al., 2001; Morest and Silver, 2003), since it is only used by neurons migrating short distances (Nadarajah et al., 2001). Accordingly, in our study the differentiated GCs traveled short distances at a slow speed (approximately 1 μm/h). In contrast, during development of the dentate gyrus, the migration speed of newly born GCs is much faster (approximately 10 μm/h), and requires a radial glial scaffold (Wang et al., 2018). Previous studies addressing migration during brain development have uncovered that the actin cytoskeleton is required for nuclear movement, involving F-actin and the myosin II motor (actomyosin), creating pulling (He et al., 2010) and pushing forces (Martini and Valdeolmillos, 2010). He et al. (2010) found that the leading tip of migrating neurons pulls the soma forward through myosin II dynamics in an actin-dependent manner, depicting a growth cone-like structure, which we did not observe. In contrast, pushing forces can be created by actomyosin contraction at the rear of the soma of a migrating neuron (Shieh et al., 2011), comparable with the results from our live cell imaging experiments. It is possible that pushing forces, acting without directional pulling forces, explain the slow migration rate and the “winding” soma of the GCs in our system.

By tracking the path of individual neurons, we could show that the majority of GCs (81.1%) moved in the direction of the hilus following a meandering path as if they were actively searching for a positional cue. In fact, Reelin-producing, hilar neurons started to degenerate already at 45 min after KA exposure and were completely lost within 4 h, resulting in an area devoid of Reelin as shown by our detailed time series of Reelin expression. Therefore, we assume that most GCs moved speci?cally to the direction of the Reelin-free hilar area, whereas only about 20% traveled in the opposite direction toward the molecular layer where Reelin-expressing neurons did not degenerate in response to KA exposure. It is possible that a functional inactivation of Reelin by impaired proteolytic processing (Tinnes et al., 2011) plays a role in the migration of GCs toward the molecular layer. Tinnes et al. (2011) showed that in KA-treated OHSC Reelin secreted by Cajal-Retzius cells is abnormally processed, leading to the production of high molecular weight Reelin unable to diffuse through the tissue and to activate enough Reelin receptors on GCs (Jossin et al., 2007; Tinnes et al., 2011).

The selective vulnerability of Reelin-positive hilar neurons has been described before in rodent epilepsy models (Gong et al., 2007; Orcinha et al., 2016) and *in vitro* in OHSC following KA exposure (Tinnes et al., 2011; Chai et al., 2014; Orcinha et al., 2016), but nobody performed such a detailed time course analysis showing death of hilar Reelin-positive cells already within 45 min after KA treatment. The observed differential survival capacity of hilar interneurons and Cajal-Retzius cells is most likely due to a lack of KA receptors on the latter, since only hilar interneurons, but not Cajal-Retzius cells, respond to KA treatment with c-Fos induction (Tinnes et al., 2011).

### Presence of Recombinant Reelin Fragments Stop KA-Induced Motility of GCs

Reelin is a key regulator of neuronal migration, delivering a signal to migrating neurons instructing them to assume their correct position during development (Tissir and Goffinet, 2003; Hirota and Nakajima, 2017; Jossin, 2020). Reelin-deficient *reeler* mice studies revealed faulty lamination in several brain structures as well as GC positioning defects in the hippocampus (D’Arcangelo et al., 1995, D’Arcangelo, 2014; Frotscher et al., 2003; Tissir and Goffinet, 2003; Zhao et al., 2004). These findings established a role for Reelin in stabilizing the lamination of the dentate gyrus. Here, we show that addition of the recombinant N-terminal Reelin fragments N-R2 and N-R6 and central Reelin fragment R3-6 to the medium of KA-treated OHSC were equally effective in preventing KA-induced migration of GCs toward the Reelin-free hilus. These data confirm and extend previous results showing that addition of the central Reelin fragment R3-6 stopped KA-induced GC migration in OHSC (Orcinha et al., 2016). R3-6 and N-R6 can activate the Reelin signaling cascade via the canonical pathway involving VLDR and ApoER2 receptors (Jossin et al., 2004; Koie et al., 2014), whereas the N-terminal fragment NR-2 has been shown to interact specifically with the extracellular domains of the EphB transmembrane proteins, inducing receptor clustering and activation of EphB signaling, independently of ApoER2 and VLDLR (Bouché et al., 2013). Reelin can also activate phosphorylation of Dab1 via binding to ephrin B proteins which are expressed on hippocampal neurons (Sentürk et al., 2011). In addition to stopping KA-induced migration of differentiated GCs, the Reelin recombinant fragments were also effective in rescuing the downstream signaling components of Reelin as shown by our quantitative Western blot analysis. They reverted the accumulation of Dab1, known to occur in the absence of Reelin (Rice et al., 1998; Bock and Herz, 2003) and reestablished the phosphorylation of cofilin, necessary for downstream effects of Reelin (Chai et al., 2009; Frotscher et al., 2017).

The effect we observed in our OHSC strongly supports the proposed hypothesis that Reelin has a positional cue function in the dentate gyrus that is impaired in the presence of KA, and small fragments derived from the Reelin molecule were capable of rescuing GCD. Similarly, the importance of Reelin for correct positioning of dentate granule cells was shown by Gong et al. (2007), who found that exogenous Reelin had a positive effect in recovering normal migration and integration of late-born GCs in dentate gyrus explants from epileptic mice. In contrast, in a recent study, quantitative analysis of interneuron-specific Reelin knockout in adult mice did not reveal structural changes in the dentate gyrus, when compared to wild type mice (Pahle et al., 2020). Interestingly, these authors showed that the number of Reelin-expressing CR cells was significantly increased in interneuron-specific knockout mice. It was proposed to be a compensatory process for the loss of Reelin-expressing interneurons experienced in these knockout mice during development, resulting in an organized GC layer. As previously shown, this compensatory mechanism is not present in the mature hippocampus, after KA treatment (Duveau et al., 2011; Orcinha et al., 2016). In our conditions, Reelin that is secreted by CR cells is abnormally processed, leading to the production of high molecular weight Reelin isoforms (Tinnes et al., 2011) unable to diffuse through the tissue. This fails to activate enough Reelin receptors present in GCs (Jossin et al., 2007; Tinnes et al., 2011), entailing migration of mature GCs and resulting in GCD. Therefore, we speculate that the observed increase in Reelin expression in the hands of Pahle et al. (2020) was enough for the maintenance of the layering in the adult dentate gyrus. In addition, in Lane-Donovan et al. (2015) GCD formation was not found after conditional Reelin knockout in the adult dentate gyrus (Lane-Donovan et al., 2015). However, in these mice, 5% of the initial Reelin concentration was still present. Given that very low Reelin concentrations (1 nM) were sufficient to produce significant effects in our study and other studies (∼ 0.5 nM; Leemhuis et al., 2010), the incomplete Reelin knockout could explain the controversy.

### Localization Matters: Placement of Reelin-Coated Beads Into the Hilus Stops KA Induced Migration

Reelin loss evidently appears to be involved in pathological neuronal migration in the adult hippocampus and substantial evidence supports the relevance of the presence of Reelin in a specific location to exert its function as a positional signal. Previous studies have shown that, during development, the rescue of GC lamination in OHSC prepared from Reelin-deficient *reeler* mice could be achieved when Reelin was present in its normal position, provided by a wild-type co-culture (Zhao et al., 2004, 2006). Here, we show that placement of Reelin-coated beads into the hilus of KA-treated OHSC could stop GC migration in a distance-dependent manner. Our real time microscopy clearly demonstrated a correlation between the localization of the Reelin source and the covered distance of eGFP-positive GCs. Neurons that were closer to the Reelin source did not significantly move away from their initial position, whereas neurons located further away from Reelin had an increased effective migration distance, but showed lower effective distances, when compared to neurons from OHSC treated with KA alone, and a directionality ratio like controls. The placement of a focal Reelin source into the hilus of OHSC was able to rescue the KA-induced movement of mature GCs in a gradient-like manner. One could explain this with the premise that Reelin fragments were produced from the full-length protein by proteolytic cleavage after Reelin was placed into the hilus due to the existence of proteases in the tissue (Jossin et al., 2007; Yasui et al., 2007). Also, the presence of small fragments bound to the beads before placement must be considered. These small Reelin fragments were then able to diffuse away from their original position, activating the Reelin signaling pathway by binding to the receptors located on the membrane of dentate GCs (Clatworthy et al., 1999; Sakai et al., 2009; reviewed in Reddy et al., 2011; Dlugosz and Nimpf, 2018; Liu et al., 2018). It is controversially discussed whether Reelin acts as a stopping signal (Dulabon et al., 2000; Chai et al., 2009; Sekine et al., 2011), as a global and locally active attractant signal (Rice and Curran, 2001; Courtès et al., 2011; Wang et al., 2018) or as a locally active repelling signal (D’Arcangelo and Curran, 1998; Wang et al., 2018) during neuronal migration in development. So far, our present results indicate that Reelin is an instructive factor, acting as a local stopping cue, responsible for maintaining the position of mature GCs and ceasing their aberrant migration under epileptic conditions.

### KA Treatment Compromises Reelin Signaling

Since Reelin can be sensed by GCs, the presence of components of the signaling cascade is imperative. To further analyze the effects of KA-induced impairment of Reelin on the behavior of GCs, expression levels of Dab1 and p-cofilin, important downstream signaling molecules of the Reelin pathway, were assessed. Reelin signaling is mediated by the ApoER2, VLDLR and EphB receptors (D’Arcangelo et al., 1999; Hiesberger et al., 1999; Trommsdorff et al., 1999; Kubo et al., 2002; Strasser et al., 2004; Bouché et al., 2013), members of the Src family of tyrosine kinases (SKF) (Arnaud et al., 2003; Kuo et al., 2005) and the neuronal phosphoprotein Dab1 (Howell et al., 1999, 2000). This pathway modulates the activity of downstream protein kinase cascades, many of which target the neuronal cytoskeleton, explaining the pivotal role of Reelin in neuronal migration. Migration involves active cytoskeleton remodeling. It includes actin filaments, microtubules and cofilin, a protein responsible for the reorganization of actin filaments. Previous studies showed that the loss of downstream signaling components lead to severe cortical alterations and perturbed hippocampal lamination, a phenotype indistinguishable from *reeler* mutants (Trommsdorff et al., 1999; Kuo et al., 2005; Bellenchi et al., 2007; Chai et al., 2009). In our study, KA treatment affected the expression of Dab1 and p-cofilin. It induced the accumulation of Dab1 and reduced phosphorylation of cofilin. However, application of recombinant Reelin fragments to KA-treated OHSC resulted in a significant decrease of Dab1 levels and increased the phosphorylation of cofilin. In a normal setting, the binding of Reelin extracellularly to its receptors induces intracellular tyrosine phosphorylation of Dab1, and thus activates other downstream components. It has been shown that mutations of Dab1 phenocopy and disruption of the Reelin signaling lead to accumulation of Dab1 protein (Howell et al., 1997; Sheldon et al., 1997; Rice et al., 1998; Hiesberger et al., 1999; Trommsdorff et al., 1999; Arnaud et al., 2003; Bock and Herz, 2003; Kuo et al., 2005), similar to what was found here after exposure to KA. Moreover, the studies from Arnaud et al. (2003) and Bock et al. (2004) reported in primary neuronal cultures that expression of Dab1 is reduced through the ubiquitin-proteasome system in the presence of Reelin and that this modification requires tyrosine phosphorylation of Dab1. In addition, Feng et al. (2007) and Franco et al. (2011) demonstrated that terminal translocation of migrating newborn neurons is Dab1-dependent. Dab1 degradation is critical in neurons to properly terminate their migration. Unfortunately, due to technical difficulties, we were not able to properly detect Dab1 phosphorylation in our system. However, these findings agree with our results showing that addition of exogenous Reelin halted neuronal migration, most likely via induction of Dab1 phosphorylation, resulting in the observed downregulation of Dab1. Reelin induces phosphorylation of Dab1, an important step for the activation of LIMK-cofilin axis. Consecutively, phosphorylation (activation) of LIMK1, a kinase that induces phosphorylation of the actin severing protein cofilin, leads to its inactivation and stabilizes the actin cytoskeleton (Arber et al., 1998; Yang et al., 1998; Meng et al., 2004; Chai et al., 2009). In an unphosphorylated state, cofilin acts as an actin-depolymerizing protein that promotes the disassembly of F-actin, leading to migration (reviewed in Frotscher, 2010). Here, p-cofilin levels were reduced in the presence of KA, and recombinant Reelin fragments abolished this effect. It is tempting to conclude that Reelin counteracts cytoskeleton reorganization and in turn stabilizes the actin cytoskeleton, stopping the migration process. In line with this argument, it was previously shown that the amounts of phosphorylated cofilin were significantly reduced in *reeler* mice and that application of recombinant Reelin to *reeler* tissue significantly increased the phosphorylation of cofilin (Chai et al., 2009, 2016; reviewed in Frotscher et al., 2017). Also, changes in cell shape during neuronal migration are associated with cytoskeletal remodeling (Rivas and Hatten, 1995; Rakic et al., 1996; Nadarajah et al., 2001; Nadarajah and Parnavelas, 2002; Miyata and Ogawa, 2007; Cooper, 2013), being characterized by well-coordinated consecutive periods of cytoskeletal stability. The reduction of phosphorylation levels of cofilin might explain the morphological changes in the soma of mature GCs observed after KA treatment in our live cell imaging experiments. Taken together, the evidences so far suggest the following scenario: after secretion, Reelin is chained to the extracellular matrix where it remains inactive until its proteolysis allows for the initiation of the downstream signaling (Lambert de Rouvroit et al., 1999; Jossin et al., 2007; Trotter et al., 2014). Reelin fragments can act locally or diffuse away from the site of secretion (Jossin et al., 2007), activating Dab1 phosphorylation by binding to its receptors present in GCs and recruiting SKFs (Howell et al., 1997; Strasser et al., 2004; Morimura et al., 2005). Phosphorylation of cofilin at serine3 renders the protein unable to depolymerize actin, thereby stabilizing the actin cytoskeleton. In the adult hippocampus, defects in Reelin-Dab1-dependent signaling caused by KA accounts for the migration of mature GCs, by somatic translocation, toward the Reelin-free hilar area, and this can be explained by the accumulation of Dab1 and the decreased phosphorylation levels of cofilin. These results point to an important role of cofilin and its coordinated regulation by Reelin-dependent phosphorylation steps in the lamination of the mature hippocampus.

### Neutralization of Reelin in the Healthy Hippocampus Dissolves GC Lamination

Additional evidence linking Reelin with the maintenance of GC lamination in the adult dentate gyrus was given by our use of the CR-50 antibody in naïve OHSC. The neutralizing antibody CR-50 can block Reelin function *in vivo* and *in vitro* (Ogawa et al., 1995; del Río et al., 1997; Miyata et al., 1997; Nakajima et al., 1997; Utsunomiya-Tate et al., 2000) and infusion of CR-50 into the hippocampus of normal adult mice induced GCD locally (Heinrich et al., 2006). Its epitope in the Reelin structure is located near the N-terminal, between the amino acids 251 and 407, important for protein homopolymerization and signaling. Mutated Reelin, which lacks a CR-50 epitope, fails to form homopolymers, and is therefore unable to transduce the Reelin signal (D’Arcangelo et al., 1997). Incubating OHSC with CR-50 caused a considerable displacement of eGFP-positive GCs in the healthy hippocampus, similar to the KA-induced phenotype. Our live cell imaging revealed that GCs migrated also by somatic translocation but, contrarily to neurons in KA-treated OHSC, in a non-meander manner, straight into one direction without significant changes in directionality, independently from their initial position within the GCL. Here, Reelin was blocked all over the OHSC. Since a positional cue was missing, the GCs immediately started to migrate without any preference in direction. These findings suggest the importance of endogenous Reelin expression and its normal topographical position for proper brain lamination. Accordingly, as previously postulated (Haas et al., 2002; Frotscher et al., 2003; Heinrich et al., 2006), Reelin is not only important for the formation but also relevant for the maintenance of GCL lamination in the adult hippocampus.

## Data Availability Statement

The raw data supporting the conclusions of this article will be made available by the authors, without undue reservation.

## Ethics Statement

The animal study was reviewed and approved by the Regierungspräsidium Freiburg.

## Author Contributions

CO performed the experiments, data analysis, and manuscript writing. AK developed the MATLAB^®^ script for data analysis. MF and EP contributed with input on data analysis. CH designed research and wrote the manuscript together with CO. All authors contributed to the article and approved the submitted version.

## Conflict of Interest

The authors declare that the research was conducted in the absence of any commercial or financial relationships that could be construed as a potential conflict of interest.

## Publisher’s Note

All claims expressed in this article are solely those of the authors and do not necessarily represent those of their affiliated organizations, or those of the publisher, the editors and the reviewers. Any product that may be evaluated in this article, or claim that may be made by its manufacturer, is not guaranteed or endorsed by the publisher.
