# Supplementary material for: Reelin Is Required for Maintenance of Granule Cell Lamination in the Healthy and Epileptic Hippocampus
Source: Front Mol Neurosci. 2021 Aug 13;14:730811. doi: 10.3389/fnmol.2021.730811 (PMC8414139; doi:10.3389/fnmol.2021.730811)
Supplement: Supplementary file 1 [file Data_Sheet_1.pdf]

Supplementary Material

Supplementary Figures

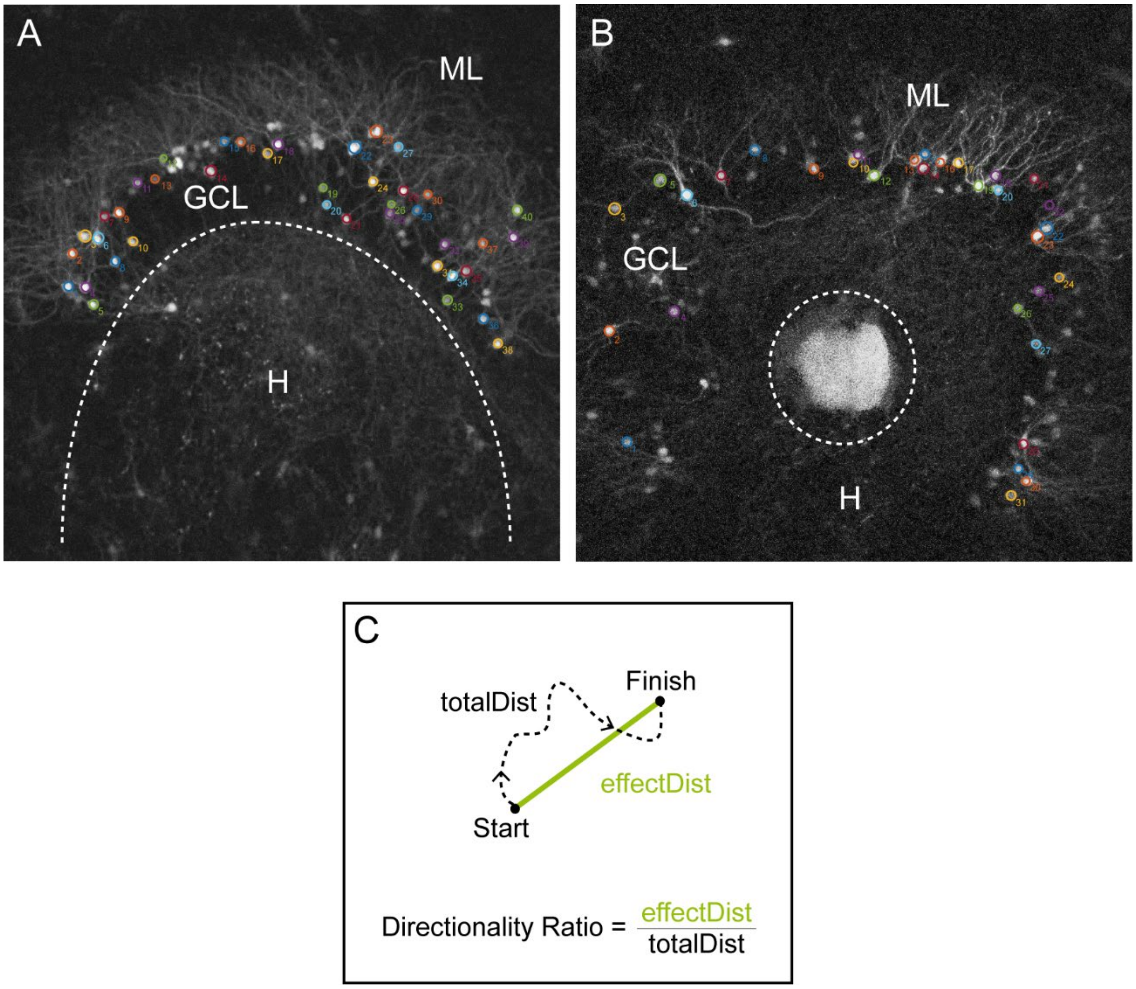

Supplementary Figure 1. Automatic evaluation of the migration behavior of individual GCs analyzed with a custom Matlab® script.
Cell bodies of the eGFP-positive GCs are automatically detected, and neurons are numbered (circles in different colors). The hilar-GCL (**A**) or microspheres (**B**) border (white dotted line, respectively) is marked by hand using an input tool implemented in the script. (**C**) Definition of the total distance (totalDist), the effective distance (effectDist) and the directionality ratio (modified from Gorelik and Gautreau, 2014). GCL, granule cell layer; H, hilus; ML, molecular layer.

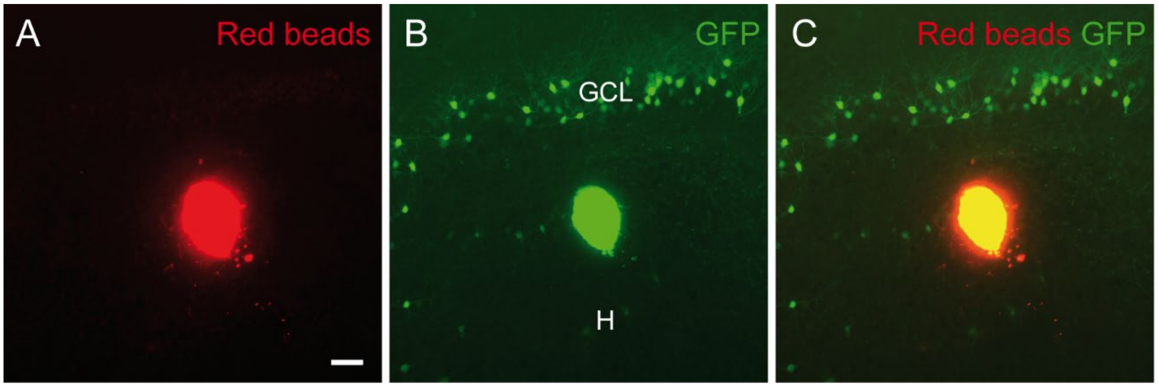

Supplementary Figure 2. Red fluorescence beads are visible in the red and green channels. Photomicrograph of the red fluorescent beads placed into the hilus (**A**). Thy1-eGFP-labeled GCs and the fluorescent beads are in green (**B**). Note the colocalization in yellow (**C**), showing that the microspheres can be visualized in both channels. GCL, granule cell layer; H, hilus. Scale bar: 50 µm.
